# Self-Assembled Nanoporous Biofilms from Functionalized Nanofibrous M13 Bacteriophage

**DOI:** 10.3390/v10060322

**Published:** 2018-06-12

**Authors:** Vasanthan Devaraj, Jiye Han, Chuntae Kim, Yong-Cheol Kang, Jin-Woo Oh

**Affiliations:** 1 Research Center for Energy Convergence and Technology Division, Pusan National University, Busan 46241, Korea; devarajvasanthan@gmail.com; 2 Department of Nano Fusion Technology, Pusan National University, Busan 46241, Korea; hanyksw20@naver.com (J.H.); chuntae1122@gmail.com (C.K.); 3 BK21 Plus Nanoconvergence Technology Division, Pusan National University, Busan 46241, Korea; 4 Department of Chemistry, Pukyong National University, Busan 48513, Korea; yckang@pknu.ac.kr; 5 Department of Nanoenergy Engineering, Pusan National University, Busan 46241, Korea

**Keywords:** M13 bacteriophage, biofilm, porous structure, filters, self-assembly

## Abstract

Highly periodic and uniform nanostructures, based on a genetically engineered M13 bacteriophage, displayed unique properties at the nanoscale that have the potential for a variety of applications. In this work, we report a multilayer biofilm with self-assembled nanoporous surfaces involving a nanofiber-like genetically engineered 4E-type M13 bacteriophage, which was fabricated using a simple pulling method. The nanoporous surfaces were effectively formed by using the networking-like structural layers of the M13 bacteriophage during self-assembly. Therefore, an external template was not required. The actual M13 bacteriophage-based fabricated multilayered biofilm with porous nanostructures agreed well with experimental and simulation results. Pores formed in the final layer had a diameter of about 150–500 nm and a depth of about 15–30 nm. We outline a filter application for this multilayered biofilm that enables selected ions to be extracted from a sodium chloride solution. Here, we describe a simple, environmentally friendly, and inexpensive fabrication approach with large-scale production potential. The technique and the multi-layered biofilms produced may be applied to sensor, filter, plasmonics, and bio-mimetic fields.

## 1. Introduction

Complex-free, cost-effective, non-lithographic, and highly ordered nanostructure fabrication approaches are necessary for diverse applications. Self-assembly methods have attracted interest because of their versatile functionalities resulting from the formations of highly ordered and well-defined nanostructures [1,2,3,4,5]. The self-assembly approach has major advantages, including large scale manufacturing capability, compatibility with experimental systems, device efficiency possibilities, and low-cost manufacturing support [6,7,8,9,10]. Highly ordered, self-assembly based nanostructures are generating interest in the filter, sensor, plasmonics, and photonics fields [11,12,13]. Despite the developmental progress, difficulties remain regarding building hierarchically ordered and highly complex nanostructures using self-assembled functional materials [14]. Understanding self-assembly and the related highly ordered complex structural arrangements can be derived from biological systems. Furthermore, biological materials and their self-assembled nanostructures can aid in the fabrication of a variety of functional nanomaterials [15,16,17,18,19,20,21]. More importantly, such fabrication methods can be eco-friendly and energy efficient [22]. Of these biological systems, viruses provide unique tools and examples of self-assembly from natural building blocks [23]. Advances in genetic engineering of viruses are providing solutions to ongoing self-assembly problems. As such, we have many opportunities to integrate viruses into various materials comprised of biologically inspired and highly self-ordered nanostructures [24,25,26,27,28].

With a well-defined geometry and flexibility resulting from modifications introduced using genetic engineering and chemical approaches, the M13 bacteriophage (M13 phage) is distinguished from other viruses because of its unique self-assembly nature that has led to a variety of novel nanostructures and devices [29,30,31]. Phages can be produced easily and cost-effectively by infecting a host bacterium, hence the name bacteriophage. After infecting a host bacterium, the host’s metabolism is adapted by the phage to continuously synthesize and secrete new phage particles, which leads to the production of millions of copies after overnight culture. The M13 phage geometry is nanofiber-like, with a diameter of approximately 6.6 nm and a height of about 880 nm, and it is covered by about 2700 copies of a major coat protein (pVIII) and five copies of minor coat proteins (pIII and pIX) located at its ends. Furthermore, due to the high fidelity of its biological reproduction, the M13 phage exhibits monodispersity and highly anisotropic shape properties. These M13 phage properties enable it to exhibit liquid crystalline (LC) behavior, and thereby form highly ordered nanostructures in suspensions. Numerous applications of the M13 phage have been reported, such as in solar cells, phage displays, imaging, biomimetic structures, photonic nose, battery, scaffolds, photo- and piezo-devices, cancer therapy, gels, filters, sensors, gene delivery, catalysis, and tissue regeneration [32,33,34,35,36,37,38,39,40,41,42,43,44,45,46].

Here, we report the fabrication of a multilayered porous biofilm consisting of alternating M13 phages and polydiallyldimethylammonium chloride (PDDA) layers for filter applications. The negative charge (E, glutamic acid) carried by the M13 bacteriophage was deliberately increased via genetic engineering to enhance its properties for this type of application. Our fabrication approach differs from those previously used to fabricate porous structures, such as previously described M13 phage-based porous structures and the breath figure method [47,48,49,50]. The differences and advantages of our fabrication from other such methods are discussed in the results and discussion section. The fabrication method used in the present study was straightforward, non-lithographic, and can be scaled up to large scale, low cost production. The formation of porous surfaces in the multi-layered biofilms produced were confirmed experimentally and by simulation results.

## 2. Materials and Methods

### 2.1. Genetic Engineering

M13 phages were purchased from New England Bio-labs (Ipswich, MA, USA) and genetically engineered using recombinant DNA methods. Using an inverse polymerase chain reaction (PCR) cloning method, the peptide sequence positioned at the 25th of the N-terminus of the wild-type phage pVIII coat proteins was engineered using PCR methods. The respective template and PCR primers were as follows: M13KE (NEB, #N0316) vector with an engineered PstI site, and the inset sequence was 5′-ATATATCTGCAGGAAGAAGAGG AACCCGCAAAAGCGGCCTTTAACTCCC-3′ respectively. The reverse primer was designed as 5′-GCTGTCTTTCGCTGC-AGAGGGTG-3′ to ensure the vector was linear and complementary to the engineered pVIII 3′-5′ region. Genetically engineered M13 phages with four glutamic (E) acids, Alu-Glu-Glu-Glu-Glu-Asp (or AEEED), were verified by DNA sequencing analysis (Cosmo-Gentech, Seoul, Republic of Korea).

### 2.2. Phage Quantification

Concentrations of M13 phage solutions were measured using an ultraviolet-visible (UV-vis) spectrometer (Evolution 300, Thermo Fisher Scientific, Waltham, MA, USA). 4E-type phage concentrations were calculated from the absorbance spectra using the previously reported formula [29,36,37]:

mg of phages/mL = (A_269_ − A_320_)/3.84

where A_269_ and A_320_ represent absorbances of a phage suspension at 269 nm and 320 nm, respectively.

### 2.3. Fabrication of Multilayer Biofilm

The biofilms were fabricated using a commercial syringe pump (LEGATO 270, KD Scientific, Holliston, MA, USA). Micro-centrifuge tubes were used to load the prepared 4E-type M13 phage and polydiallyldimethylammonium chloride (PDDA) solutions. The concentrations of the 4E-type M13 phage and PDDA solutions were 5 mg/mL and 2 wt %, respectively. The glass substrate was attached to metal tweezers. The syringe pump’s built-in software was used to control dipping and pulling speeds. Our biofilm consisted of 10 layers, 5 layers each of M13 phage and PDDA deposited in an alternating manner. Oxygen (O_2_) plasma was used to clean the glass substrate and imbue hydrophilicity, which was then dipped in cysteamine to facilitate cross-linking with the M13 phage. To produce the multilayers, the glass substrate was dipped in the respective solutions for 3 s to form each layer. The glass substrate was pulled out from solution(s) after every layer deposition and left to dry naturally at room temperature for 30 min. The processes were repeated until all 10 layers had been deposited.

### 2.4. Structural Characterization

AFM images were collected using an NX10 unit (Park Systems, Suwon, Korea) equipped with the XEP 3.0.4 data acquisition program (Park Systems). Obtained AFM images were analyzed using the XEI 1.8.2 image processing program (Park Systems). All images were collected in true non-contact mode. A specialized probe for non-contact mode was used for measurements (PPP-NCHR, Nanosensors, Neuchatel, Switzerland). Optical images of M13 phage/PDDA multi-laminated films were obtained by scanning electron microscopy (SEM; S4800, Hitachi, Tokyo, Japan).

### 2.5. Analysis of Adsorbed Ions on Multilayer Biofilms

The thin film surface elements were analyzed by X-ray photoelectron spectrometry (XPS; ESCALAB 250, Thermo Fisher Scientific, Waltham, MA, USA) equipped with a hemispherical analyzer and a twin anode non-monochromatic Al-Kα source. For selective ion filter application, two types of films were prepared on glass slides: M13 phage only thin film and M13 phage/PDDA film. These films were immersed in NaCl solutions with different concentrations (0, 0.1, 0.2, 0.5, and 1 M) to bind ions, washed in distilled water to remove excess ions, and dried. Ions bound on surfaces in films were then analyzed by XPS.

### 2.6. Theoretical Method

Reflectance simulations of multilayer biofilms were performed using three-dimensional (3D) finite-difference time-domain (FDTD) simulations using a commercial package from Lumerical Solutions (Vancouver, BC, Canada). The multilayered biofilm structure was surrounded by a perfectly matched layer (PML) boundary conditions in the *x*, *y*, and *z* directions. To obtain accurate results, λ/400 elements of mesh size were applied. A broadband plane wave source was used for optical excitation, and a power monitor was placed at top of the multi-layered biofilm structure in the air region to record reflectance. At λ = 600 nm, refractive indices *n* of the slide glass, the M13 bacteriophage, and the PDDA were 1.514, 1.45, and 1.375, respectively [51].

### 2.7. Reflectance Experiment

Reflectance measurements were recorded using a Thermo Scientific Evolution 300 UV-Vis spectrophotometer, a xenon lamp, high resolution (1200 lines/mm) grating, and a silicon photodiode detector.

## 3. Results and Discussion

**Figure 1:**
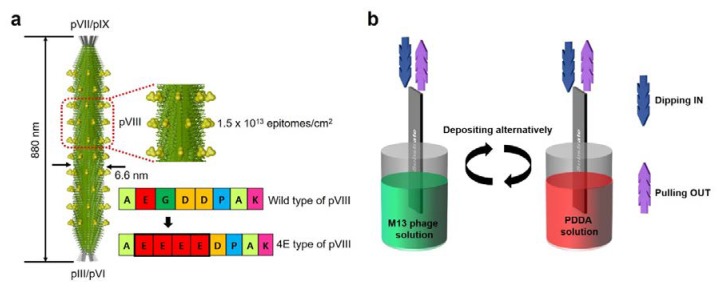
(**a**) Illustration of the structure of M13 phage and pVIII, pVII/pIX, and pIII/pVI amino acid sequences. pVIII genetic engineering site information shows conversion from wild type to 4E-type phage. (**b**) Schematic of the pulling method used to produce M13 phage/polydiallyldimethylammonium chloride (PDDA) multilayered films on glass.

**Figure 2:**
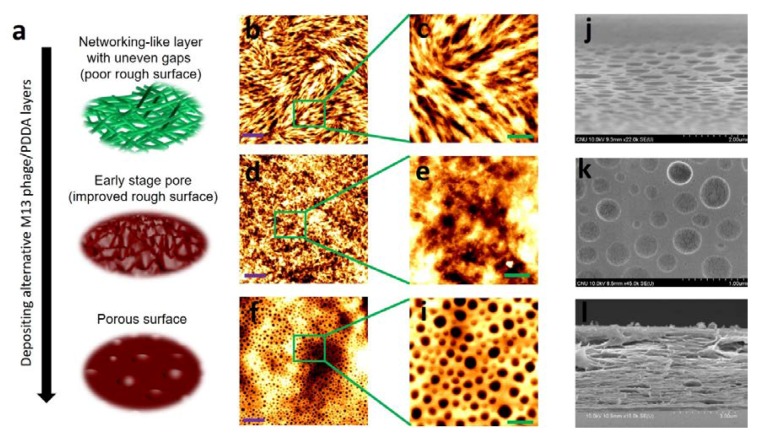
(**a**) Fabrication of porous surfaces by depositing M13 phage and PDDA layers in an alternating manner originating from a randomly distributed network-like structure. (**b**,**c**) AFM images of first layer, (**d**,**e**) sixth layer, and (**f**,**i**) 10th layer surfaces. Violet and green scale bar colors represent five and one µm, respectively. Scanning electron microscopy (SEM) images taken from a 10-layer biofilm: (**j**) perspective view, (**k**) top view, and (**l**) cross-sectional view. Scale bars for (**j**–**l**) are two, one, and three µm, respectively. The cracks shown in figure (**k**) were caused by platinum coating for SEM analysis.

Information on the geometry of the M13 phage is provided in Figure 1a. The M13 phage has a nanofiber-like structure about 880 nm long with a diameter of about 6.6 nm. The nanofibrous M13 phage contains about 2700 copies of pVIII amino acid sequences with pIII, pVI, PVII, and pIX amino acid sequences at its ends. As described earlier, multi-layer nanoporous biofilms were fabricated by pulling method (PM) using commercial syringe setup. A PM fabrication schematic is shown in Figure 1b. A glass slide (the substrate), attached to a commercial syringe pump, was dipped in and then removed from M13 phage and PDDA solutions in an alternating manner. The mechanism used to form nanoporous surfaces in biofilms involves three steps: (1) deposition of initial layers with poor quality rough surface quality based on initial M13 phage networking layer(s), (2) improve surface quality by depositing additional layers leading to early stage pore formation, and (3) to form nanoporous surfaces in final layers (Figure 2a).

To create the initial layers, the flexible nature of the geometry of the M13 phage plays a crucial role. During initial deposition, M13 phages are deposited randomly to form a nanofiber network, which results in a poor quality and uneven surface (Figure 2b,c). This network structure can be achieved by varying deposition time, speed, and M13 phage concentration. More importantly, these initial layers are made possible by the liquid crystalline (LC) behavior of M13 phages during self-assembly. When the substrate was subjected to PM, evaporation occurred more rapidly at the air-liquid-solid meniscus, which resulted in the local accumulation and deposition of M13 phages on the substrate. At this time, two crucial factors are thought to occur during self-assembly: local induction of chiral LC structure phase transitions occurring at the meniscus, and dominance of interfacial forces acting at the meniscus. By controlling the conditions mentioned above during biofilm growth, producing different self-assembled nanostructures is possible. LC phases are classified as thermotropic or lyotropic. Of these, the lyotropic phase plays a crucial role in the self-assembly of biological structures. By varying temperature and concentration of biological particles, producing lyotropic LCs with many phases is possible. Of the experiment conditions, the most critical factor is concentration (of M13 phage solution in this study), which dominantly affects the lyotropic LC phase. To fabricate a biofilm layer with a network structure with an uneven surface, we prepared M13 phage solution at a low concentration (five mg/mL). At this low concentration, M13 phages were distributed randomly on the substrate, that is, an isotropic phase. Such conditions satisfy the needs of the initial layers with a poor surface quality with a surface roughness of ~70 nm.

PDDA films were then deposited in an alternating manner on the M13 phage layers. At this point, the role of PDDA layers was to reduce the surface roughness of previous M13 phage layers. Notably, uneven gaps created by poor surfaces in the network structures ranged from about 1 to 3 µm. These gaps in the layers were gradually filled by depositing PDDA layers, and when around six layers had been deposited, early stage pore formation was observed (Figure 2d,e) with better surface quality. The surface roughness was now ~37 nm, which is an improvement of about 50%. However, at this stage, a few surface nanopores with diameters of about 700–1200 nm (±20 nm) and depth of ~70–80 nm (±5 nm) were evident. Further depositions of M13 phage and PDDA layers improved surface quality and induced the formation of pores with decreasing diameters and depths. Clear porous surfaces were seen after depositing the eighth layer. The porous surfaces eventually formed by the 10th layer are shown in Figure 2f,i. Pore diameters ranged from ~150 to 500 nm (±20 nm) and depths from ~15 to 30 nm (±5 nm) in the 10th layer. The surface quality of the final layer improved by about 80% when compared with the first layer (surface roughness of ~14 nm). This porous surface information was well supported by the perspective (Figure 2j) and surface view (Figure 2k) of the SEM images. After 10 layers, clear porous structures were observed with diameters ranging from ~150 to 500 nm. The total thickness of the 10-layered bio-film was ~3.84 µm (SEM cross-sectional view, Figure 2l).

We then considered two porous structure fabrication concepts: breath figure (non-M13 phage-based structures) and M13 phage-based porous fabrication methods [47,48,49,50,52]. With the breath figure method, careful optimization of experimental parameters is required to achieve highly ordered porous structures. Furthermore, the open experimental process to fabricate porous structures is difficult. For example, in static breath figure methods, fabrication processes were sealed from external environmental lab conditions. Generally, in the breath figure process, water droplets formed from condensation, acting as a template to create a porous structure. Condensation of water vapours is vital in the breath figure process. Careful steps should be considered to prevent the water droplet template from effects such as a coalescence. Methods reported involving a combination of dip coating, similar to our pulling method, and the breath figure only showed porous structure formation in the presence of an external mesh template, such as nylon mesh [48,52].

When using fabrication methods involving M13 phages, porous structures were successfully formed either based on a supporting macroporous film, like anodic aluminum oxide (AAO), or by the freeze-drying method [49,50]. Nanoporous structures with diameters of about 200 nm formed using AAO template also used the etching process. In previously reported layer-by-layer biofilms involving M13 phages, ordered networking structures were formed but no nanoporous surfaces were reported [53]. Please note, when a wild type M13 phage was used instead of 4E type, networking structures were observed but no nanoporous surfaces were formed in the multilayer biofilm. Biofilms involving 4E type phage formed nanoporous surfaces successfully due to repulsive charge properties based on our fabrication method. Our fabrication method was simple, straightforward, and can be performed in an open, room temperature lab conditions. More importantly, our method does not require an external template to form a nanoporous structure. Our method is non-lithographic, and thus no etching process is required. Additionally, large scale fabrication is possible at comparable low costs. Given these advantages, our approach can facilitate the fabrication of nanoporous structures and might open interesting possibilities for a variety of applications, such as separation, bio-mimetic structures, plasmonics, sensors, etc. To justify our fabrication approach, we performed an optical investigation that confirms the structural transitions.

### 3.1. Optical Results: Theoretical and Experimental

We confirmed structural transitions of biofilms from poor surface quality to the porous surface using three-dimensional FDTD reflectance simulations and then performed experimental verification. Topographic information for each layer obtained from AFM data was used to create a layer geometry in FDTD. The biofilm structure was modeled as follows: no porous surfaces occurred during the first five layers, and after that, porous surfaces were created during the subsequent layers (6–10 layer). Surface roughness data obtained by AFM was found to be useful for creating uneven surfaces. We modeled the pore shape as parabolic one using the following equation [54]:

(1) $$z=cx^{2}$$

**Figure 3:**
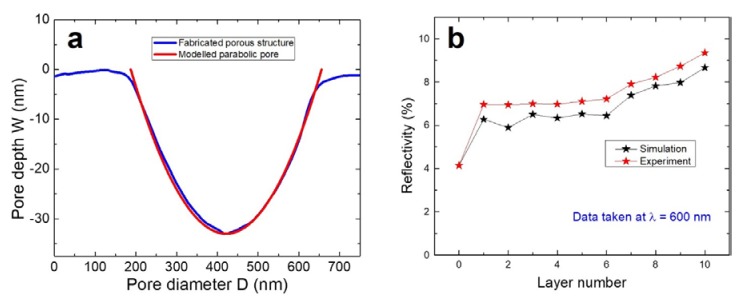
(**a**) Parabolic-shaped pore depth versus diameter plot obtained using Equation (1) (*z* = *cx*^2^) aligns well with observed pore shapes as determined using the line profile AFM data for a 10-layer biofilm. (**b**) Experimental and modeled reflectance values obtained at λ = 600 nm for a 10-layer biofilm.

The term “*c*” is defined by:

(2) $$c=4\times \;\frac{D}{W^{2}}$$

where *D* and *W* are pore diameter and depth, respectively (Figure 3a). At λ of 600 nm, no absorbance issues were observed for the glass substrate, M13 phage, or PDDA layer. This wavelength data will help analyze the reflectivity, as no other complex optical information is related to these three materials. Figure 3b shows the simulated and measured reflectivity data as function of layer number. Layer number 0 corresponds to the glass substrate. The reflectance of 4.2% is typical of glass substrates, and this well-matched the experiment and simulation results. From layer numbers 1 to 6, almost no difference was observed in reflectance data. At layer 1, a poor surface morphology increased the reflectance to ~6.5%, and up to layers 5 or 6, similar reflectance values were recorded. Furthermore, this reflectance concurred with experiment results, with average reflectance values of ~7% being measured for layers 1–6. Significant reflectance changes were observed from layer 7 to 10. At this point, the influence of the porous surface played crucial role. Reflectance values increased from ~7% to ~9%, due to the presence of porous nanostructures in the surface. For a 10-layered biofilm, ~8.7% reflectance was obtained via simulation. Experimental data showed a similar trend for reflectance values and a maximum value of ~9.5% was recorded for a 10-layer biofilm. This linear increase in reflectivity values can be explained using a solid immersion lens (SIL) structure. Our pores were shaped like an inverted SIL, which is parabolic shaped [54]. For pores with larger diameters and depths, light could be more easily transmitted, comparatively. With these dimensions, internal diffraction and reflection can be minimized resulting in a smaller reflectance value. At smaller pore dimensions (smaller diameter and depth), contributions from internal diffraction and reflections increase, resulting in an increase in reflectivity. These optical properties aligned well with our simulation and experimental results and support the proposed fabrication mechanism responsible for surface pore formation arising from initially poorly deposited layers and the presence of randomly distributed network-like structures. This approach opens an interesting path for the fabrication of self-assembled porous multilayer biofilms.

### 3.2. Filter Experiment

**Figure 4:**
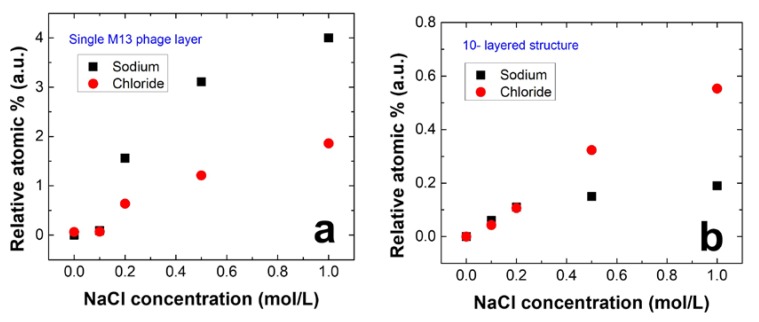
Calculated relative atomic percentages obtained from X-ray photoelectron spectroscopy (XPS) measurements for (**a**) M13 phage only and (**b**) 10-layer M13 phage-PDDA nanoporous biofilms, which confirm selective ion filtration.

To investigate potential applications of the M13 phage and PDDA laminated nanoporous structure as a functional filter, we examined its ability to extract ions from sodium chloride (NaCl) solution. For this purpose, two types of films were tested: a M13 phage only film and a M13 phage-PDDA multilayered nanoporous film. Figure 4a,b show the relative atomic ratios of the captured ions at different NaCl solution concentrations for the two films. In the absence of a PDDA layer, Na^+^ ions were captured more effectively by the negative charges of M13 phages. However, in the M13 phage-PDDA multilayer biofilms, a larger number of Cl^−^ ions were captured by the positively charged PDDA. Although the laminated structure consists of the M13 phage, Na^+^ ions that permeated into pores and bound to virus layers were not detected because of the limited skin depth of the XPS system. This result indicates that Cl^−^ ions bound effectively to the positive charges of PDDA in the multi-layered biofilm. Eventhough the capture ratio of the Cl^−^ ions was smaller at this time, further improvements will be performed in the near future. Problems arise when performing selective ion extraction experiments on a single-layered PDDA film. The success rate of PDDA film attached to the substrate when dipped into NaCl solution was ~ < 1%. Thus, we believe the ten layered biofilm is far better way to proceed. Finally, our results confirm that multi-layer biofilms with a nanoporous structure can act as membranes for selective ion extraction.

## 4. Summary

In summary, we reported a straightforward fabrication approach to produce nanoporous multilayered M13 phage-PDDA films using a pulling method. Our fabrication approach was non-lithographic, and nanoporous structures with diameters of about 150 nm were formed without the support of an external template. Good agreement between experiment and simulation results support our suggested fabrication mechanism. M13 phage nanofiber geometry played a significant role in pore formation during our fabrication process. The described process has the advantages of straightforward fabrication, low-cost manufacturing, and compatibility with large-scale production. The devised nanoporous multi-layer biofilm provides a novel option for selective ion filtration, and may have interesting applications in the fields of plasmonics, photonics, and sensors.
